# Supplementary material for: MetaRibo-Seq measures translation in microbiomes
Source: Nat Commun. 2020 Jun 29;11:3268. doi: 10.1038/s41467-020-17081-z (PMC7324362; doi:10.1038/s41467-020-17081-z)
Supplement: Supplementary file 10 — Supplementary Data 7 [file 41467_2020_17081_MOESM10_ESM.zip › File2/Confidence_VeryHigh_Taxonomy/222488_out.krona.html]

Javascript must be enabled to view this page.

members
magnitude
magnitudeUnassigned
count
unassigned
taxon
rank

222488\_out

9

1

SRS016989\_contig\_number\_contig-100\_10245.46767

8
2
superkingdom

1239
phylum
8

186801
class
8

order
186802
8

family
186806
1

1730
genus
1

39485
species

SRS146812\_contig\_number\_59411
1

6
186803
family


SRS013687\_contig\_number\_14342SRS015794\_contig\_number\_contig-100\_3580.3580SRS019068\_contig\_number\_116317SRS019445\_contig\_number\_9285SRS075078\_contig\_number\_15751
5
39491
species

1
genus
841


SRS015431\_contig\_number\_61387
1
360807
species

family
31979
1

1485
genus
1

2293011
species

SRS023715\_contig\_number\_20691
1
